# Peripheral Osteoma of the Subcondylar Region in the Mandible Treated Using the High Perimandibular Approach: A Case Report

**DOI:** 10.1155/crid/8830675

**Published:** 2025-03-15

**Authors:** Tamaki Sekiguchi-Yamada, Kazuhiro Matsushita, Rieko Yoshitatsu, Aya Yanagawa Matsuda, Tomoka Hasegawa, Yoichi Ohiro

**Affiliations:** ^1^Oral and Maxillofacial Surgery, Department of Oral Pathobiological Science, Faculty of Dental Medicine and Graduate School of Dental Medicine, Hokkaido University, Sapporo, Hokkaido, Japan; ^2^Stomatognathic Function, Center for Advanced Oral Medicine, Hokkaido University Hospital, Sapporo, Hokkaido, Japan; ^3^Vascular Biology and Molecular Pathology, Faculty of Dental Medicine and Graduate School of Dental Medicine, Hokkaido University, Sapporo, Hokkaido, Japan; ^4^Developmental Biology of Hard Tissue, Graduate school of Dental Medicine, Hokkaido University, Sapporo, Hokkaido, Japan

## Abstract

Osteomas are benign tumors composed of mature, compact, or cancellous bone that commonly arise in the sinuses and exhibit slow and asymptomatic growth. Surgical resection may be performed using intraoral or extraoral approaches when functional or morphological deficits are present. A 63-year-old female presented with an osteoma in the subcondylar region, which had doubled in size 9 years after the initial diagnosis. Considering the potential risk of functional impairment, the osteoma was excised using the high perimandibular approach. Two years postsurgery, the patient remained asymptomatic, with no functional or morphological deficits or recurrence of the disease.

This case report describes the successful use of the high perimandibular approach for managing an osteoma in the subcondylar region and highlights its clinical versatility.

## 1. Introduction

Osteomas in the subcondylar regions, as observed in this case, are extremely rare. We encountered a case of peripheral osteoma in the subcondylar region, which we monitored for 10 years. Over time, the tumor grew so large that intraoral excision was no longer feasible, resulting in conspicuous facial asymmetry. Among the various extraoral approaches, the high perimandibular approach was selected despite being originally developed for fracture treatments. This technique provided excellent intraoperative surgical field visualization, facilitating efficient handling. The postoperative outcome was highly satisfactory, demonstrating that this approach is also effective for the surgical excision of peripheral osteomas in the subcondylar region. Here, we present a summary of the case along with our observations.

## 2. Case Presentation

**Figure 1:**
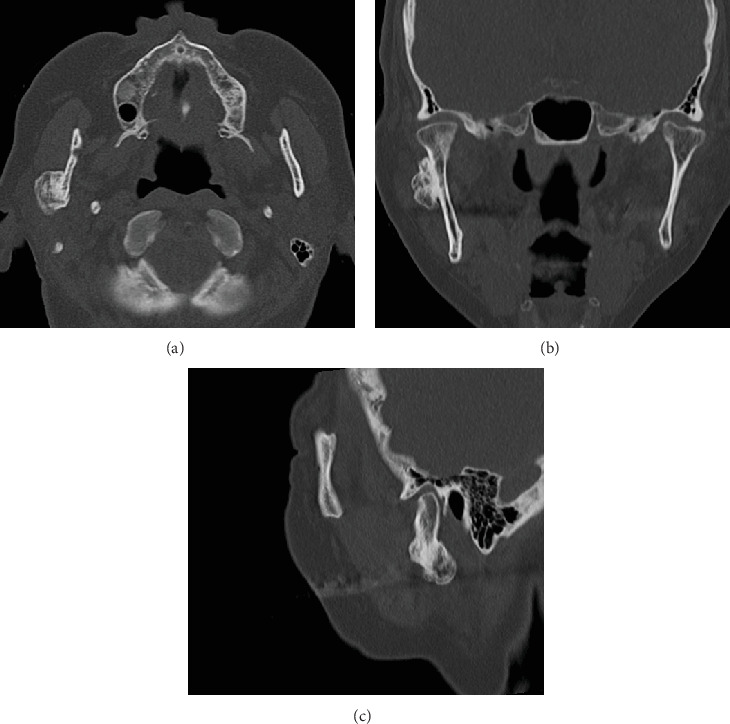
(a) Axial section of the computed tomography scan. (b) Coronal section of the computed tomography scan. (c) Sagittal section of the computed tomography scan.

**Figure 2:**
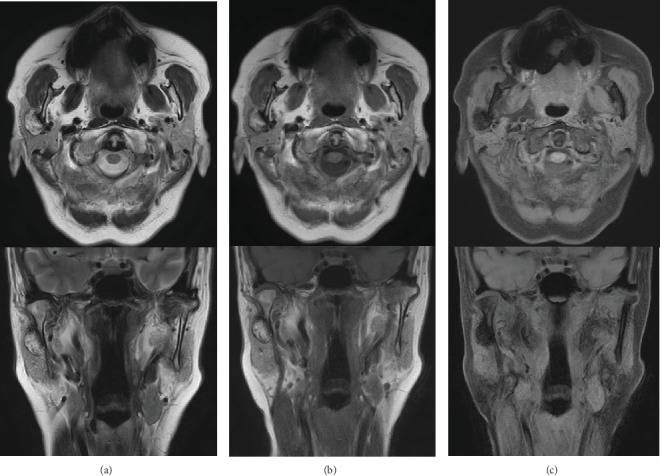
(a) Top: axial section of the magnetic resonance imaging (MRI) scan on T1. Bottom: coronal section of the MRI scan on T1. (b) Top: axial section of the MRI scan on T2. Bottom: coronal section of the MRI scan on T2. (c) Top: axial section of the MRI scan on T1 with fat suppression. Bottom: coronal section of the MRI scan on T1 with fat suppression.

**Figure 3:**
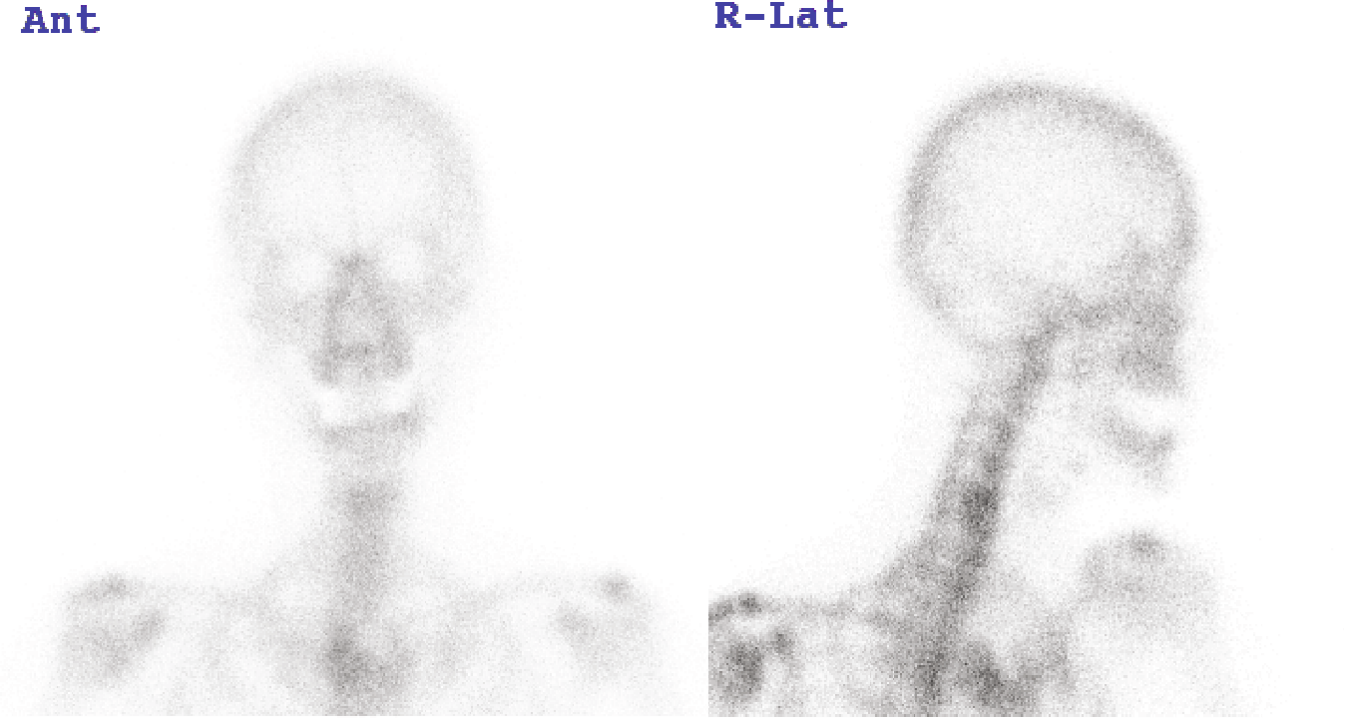
Bone scintigraphic finding with ^99m^Tc-MDP.

**Figure 4:**
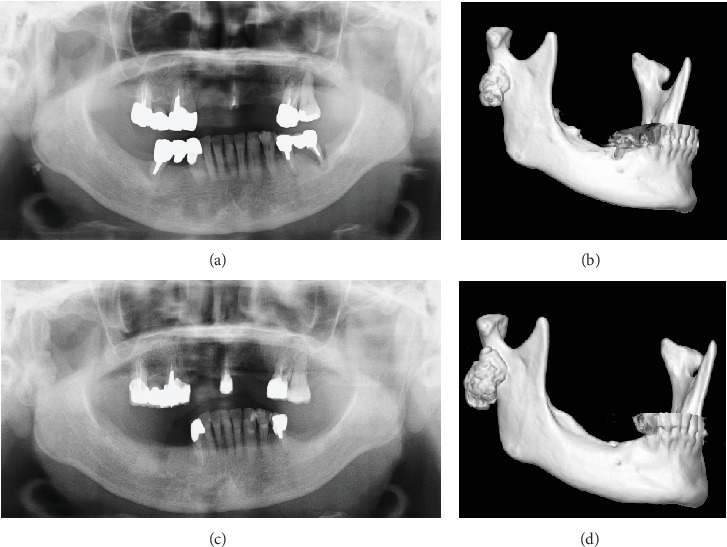
(a, c) Orthopantomography and (b, d) three-dimensional computed tomography images reveal a bony lesion at the right subcondylar region of the mandible. (a, b) The first visit. (c, d) Nine years after the first visit.

**Figure 5:**
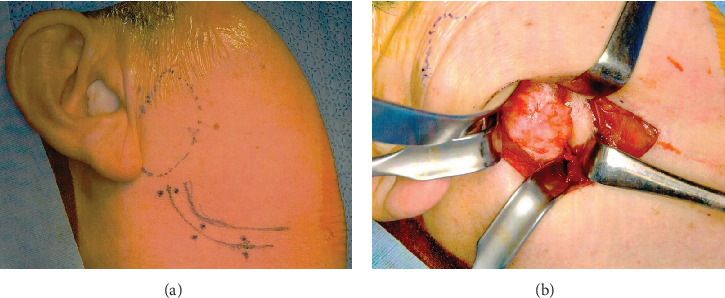
(a) The incision line of the high perimandibular approach. (b) Intraoperative view of the lesion exposed by the high perimandibular approach.

**Figure 6:**
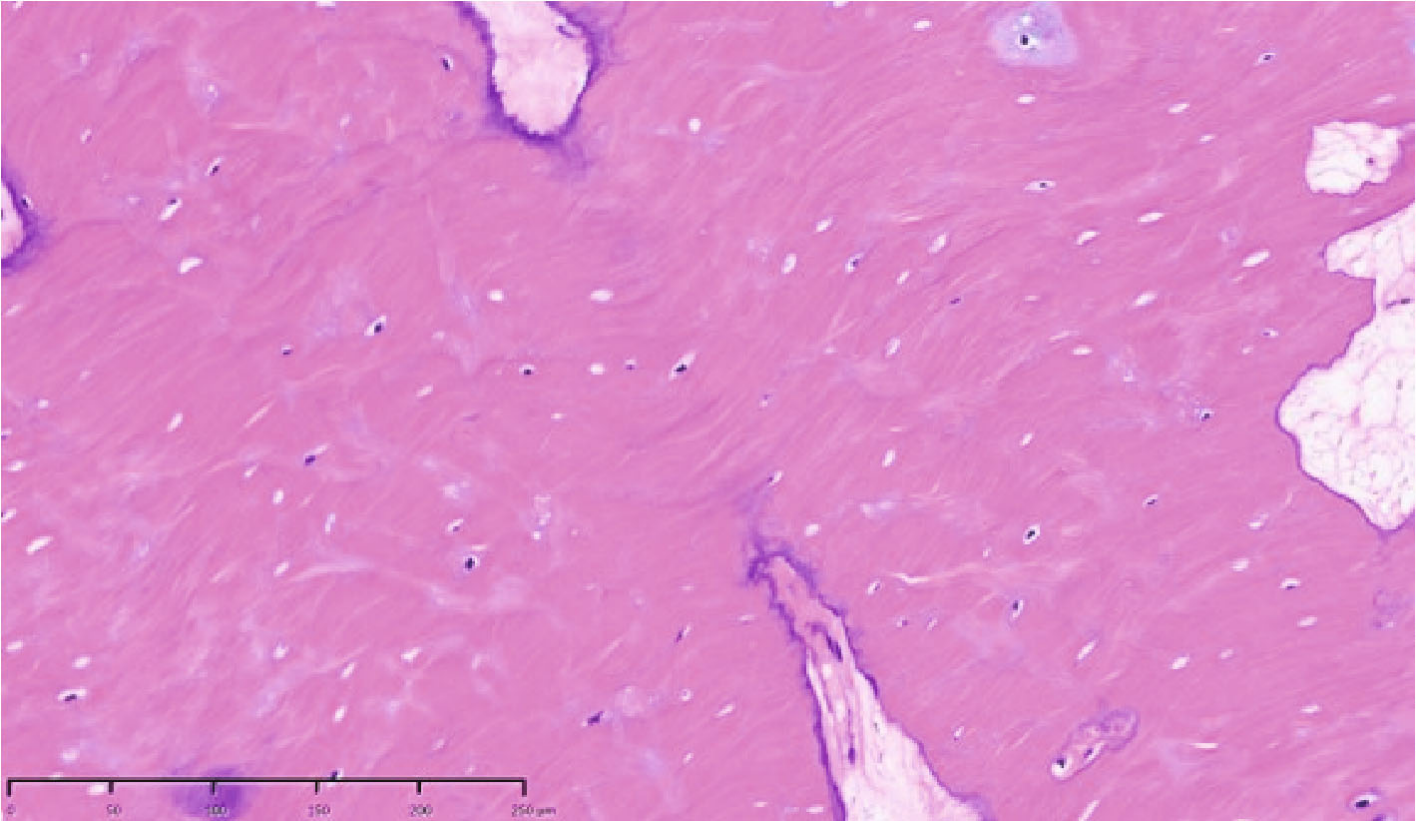
Hematoxylin and eosin staining of the surgical specimen. Dense cortical bone showing laminar structure and trabecular bone in the form of beams. Osteocytes are small and show little nuclear atypia or mitotic figures. × 20, bar = 50 *μ*m.

**Figure 7:**
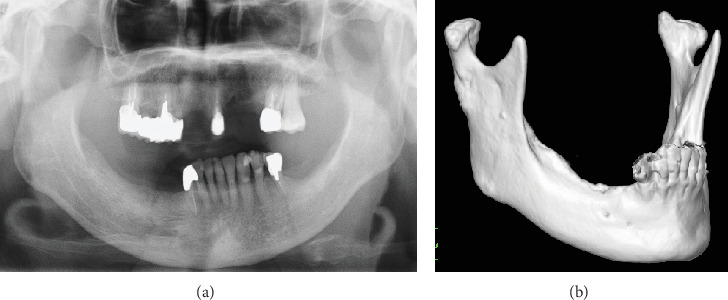
(a) Orthopantomography and (b) three-dimensional computed tomography image at 2 years postoperatively.

A 63-year-old woman was referred to our department due to a hard, immobile mass in the right parotid region with a masseteric extension in 2012. She reported no pain or functional impairment. Orthopantomography revealed a morphologically irregular opacity overlapping the posterior border of the mandibular ramus. Computed tomography demonstrated a lesion with bone-like high resorption values contiguous with the mandible. The margin of the lesion appeared to be composed of thin bone, whereas the internal contained a heterogeneous bone beam-like structure (Figures 1a, 1b, and 1c). Magnetic resonance imaging identified an irregular mass partially contiguous with the mandible. On both T1- and T2-weighted images, parts of the mass exhibited high-intensity signals (Figure 2a,b), while most areas demonstrated signal reduction with fat suppression (Figure 2c). Bone scintigraphy using ^99m^Tc-MDP/HMDP confirmed no increased blood flow in the perfusion phase and no hyperaccumulation in the bone phase (Figure 3). Despite the evident facial asymmetry, the patient remained asymptomatic. Based on these findings, a clinical diagnosis of osteoma was made, and we opted for annual follow-up observations as management. The patient was followed up annually for 5 years but was then lost to follow-up. Four years later (9 years since the initial consultation), she returned to our clinic. Although still asymptomatic, the lesion had doubled in size (Figures 4a, 4b, 4c, and 4d). Given the proximity of the lesion to the condyle and the potential for future jaw dysfunction, surgical excision was deemed necessary. Among the various methods, we opted for the high perimandibular approach [1]. The procedure was performed following the standard protocol (Figure 5a). The lesion was well-defined (Figure 5b) and was removed using rotary instruments, chisels, and ultrasound bone-cutting equipment. Pathological analysis showed dense cortical bone with a laminar plate structure and trabecular bone. The osteocytes were small, without evidence of nuclear atypia or mitotic activity (Figure 6). The histopathological examination revealed osseous tissue compatible with that of an osteoma. No facial paralysis, wound infection, or scarring was observed postoperatively. The patient's range of mouth opening was unchanged, and no recurrence has been observed over 2 years since the surgery (Figure 7a,b).

## 3. Discussion

Osteomas are classified into central, peripheral, and extraskeletal types [2]. Central, peripheral, and extra-skeletal soft-tissue osteomas develop from the endosteum, periosteum, and muscle tissue, respectively. Clinically, peripheral osteomas typically present as unilateral, sessile, pedicled, well-circumscribed, mushroom-like masses, ranging from 10 to 40 mm in diameter [3]. They can occur at any age and show no sex prediction [4]. Although the exact etiology and pathogenesis remain unclear [5], trauma, inflammation, infection, and muscle traction are suspected contributing factors [3]. The clinical course and imaging findings in this case indicated a typical peripheral osteoma; however, the exact cause of its development remains unclear.

These tumors commonly occur in the craniofacial bones, such as the paranasal sinuses, orbital walls, temporal bones, pterygoid processes, and external auditory canals, but are rarely observed in the jaw [6]. If present in the mandible, they most frequently affect the posterior body, followed by the condyle and angle [7].

Peripheral osteomas that grow externally are usually asymptomatic because of their limited size. If they do not cause significant aesthetic or functional impairments, conservative management is recommended. However, when the lesions become large enough to cause symptoms, as in our case, surgical intervention is necessary. The choice between the intraoral and extraoral approaches should be based on factors such as lesion size, location, surgical safety, and postoperative aesthetics [8].

Although several reports on peripheral osteomas of the mandible have been published, only eight cases involving the subcondylar region have been documented [9]. Among these, three cases were treated using the intraoral approach, one using the extraoral approach, and details for the remaining cases were unspecified [9]. Surgical treatment for osteoma is necessary when the lesion is symptomatic and progressive, as peripheral osteoma in the jaw can increase in size and cause significant damage. The choice of intraoral or extraoral approach should be based on the safety of the surgical field and the feasibility of complete resection. The use of the intraoral approach reduces postoperative scarring and the risk of facial nerve injury. However, avoiding damage to vital structures, such as the zygomatic branch of the facial nerve, maxillary artery, and mandibular neurovascular bundle, is challenging. Depending on the osteoma size and location, the surgical field may be obstructed by the coronoid process. Sometimes, extensive mucosal incision or coronoid resection is required, increasing the risk of fracture or injury to the surrounding tissues. Literature reports reveal that the intraoral approach was chosen when the osteoma size and location allowed safe instrumentation and complete removal with minimal tissue damage. However, in one report, the extraoral approach was performed because the base of the osteoma made it difficult to access the condylar neck intraorally. Considering these findings and the fact that the lesion in our case extended medially beyond the posterior edge of the mandible, we opted for an extraoral approach for the surgical excision.

Several extraoral approaches have been described for accessing the mandibular condylar and ramus regions, such as the preauricular, submandibular, retromandibular, and high-perimandibular approaches [10–12]. The high perimandibular approach was first described by Wilk [1] and has gained popularity for managing mandibular condylar fractures because of its excellent visualization of the fracture site while minimizing the risk of facial nerve injury. This technique offers the significant advantage of reduced postoperative scarring compared to traditional extraoral approaches. We hypothesized that the high perimandibular approach is effective for treating fractures and various lesions in the subcondylar region.

The selection of the surgical approach depends on the location and nature of the lesion, as well as the course of the facial nerve, which is a critical factor. Among the branches of the facial nerve, special attention must be given to the marginal mandibular branch because of its limited networking with other branches and its relatively high risk of injury, which can lead to functional disturbances in 0%–16% of cases [11]. The reported incidence of motor nerve disturbances following the Risdon and retromandibular approaches is 11%–30% and 7%–47%, respectively [13]. In contrast, studies have reported a significantly lower incidence of facial nerve disturbances (0%–0.9%) with the high perimandibular approach, making it a preferable technique for preserving nerve function [13]. In our case, we selected the high perimandibular approach to ensure the preservation of the marginal mandibular branch while achieving direct intraoperative visualization of the subcondylar region. According to previous reports, this approach has also been successfully employed in nonfracture cases, such as the excision of intramasseter tumors with no facial nerve damage and minimal postoperative scarring [13].

The high perimandibular approach in our case allowed for optimal visualization of the base of the lesion on the lateral surface of the ramus. By viewing the surgical field from underneath rather than from above, we achieved a clearer surgical perspective. The osteoma extended medially beyond the posterior edge. We extended the dissection slightly above the platysma muscle in the posterior direction to enhance visualization. We then performed a precise incision in the masseter. This approach facilitated layer-to-layer elevation and enabled clear identification and preservation of the buccal branch of the facial nerve running along the masseter fascia, thereby minimizing the risk of nerve injury.

No postoperative facial paralysis was observed. Our findings highlight the high perimandibular approach as a highly effective technique for the removal of subcondylar lesions, including osteomas.

## 4. Conclusion

We present a case of peripheral osteoma in the mandibular subcondylar region. The high perimandibular approach proved to be an effective surgical technique, providing a wide, unobstructed field while significantly reducing the risk of facial nerve damage, particularly to the marginal mandibular branch. Although osteomas do not undergo malignant transformation, reported cases of recurrence necessitate long-term postoperative follow-up.

## Data Availability

All data related to the presented case are included in this published article.
